# Long-Term Comparison between Waste Paper Fly Ash and Traditional Binder as Hydraulic Road Binder Exposed to Sulfate Concentrations

**DOI:** 10.3390/ma15155424

**Published:** 2022-08-06

**Authors:** Hani Baloochi, Marilda Barra, Diego Aponte

**Affiliations:** Department of Civil and Environmental Engineering, Universitat Politècnica de Catalunya (UPC-BarcelonaTech), Jordi Girona 1-3, 08034 Barcelona, Spain

**Keywords:** waste paper fly ash (WPFA), soil stabilization, sulfate attack, long-term swelling, ettringite formation

## Abstract

Sulfate attack is one of the drawbacks of cementitious materials for stabilized soils. In the current study, a durability comparison of stabilized soil with cement (Type IV) and waste paper fly ash (WPFA) was conducted. First, the treated soil’s unconfined compressive strength (UCS) was tested. Next, the treated soil was subjected to various wetting/drying cycles with various sulfate concentrations and temperatures for a year. In the meantime, samples were taken for DRX, FTIR, and TGA microstructural analyses. Additionally, samples were manufactured to track swelling over an 800 day period. The outcomes show that WPFA’s UCS remained constant. Furthermore, ettringite development can be seen in the microstructural studies, however testing on linear displacement over 800 days revealed no significant changes in swelling. Finally, SEM was used to verify the ettringite formation at 360 days in order to confirm the previous findings. All the results indicated that stabilizing soil with 5% of WPFA and 3% of cement IV is possible even in presence of high sulfate concentrations, while maintaining the durability of the structure.

## 1. Introduction

The most common way to stabilize a soil is with the use of binders, such as lime and cement [1,2,3,4,5,6]. These binders improve the strength and workability of the soils via ion exchange or by forming C–S–H gel and calcium carbonate [4]. One of the disadvantages of using these binders is sulfate attack. Sulfate attack in concrete has been studied vastly in the concrete sector [7,8,9], and it is well defined as the reaction between sulfate and certain compounds in concrete that leads to the expansion and formation of cracks in concrete. To be specific, sulfate in certain conditions reacts with cement compounds such as monosulfate, portlandite and C–S–H gel. The results of this reaction may be ettringite, gypsum, or thaumasite [10,11].

However, there are two completely different opinions regarding the use of these binders in soils. The first is that these expensive materials in soil are beneficial and can fill up the pores in soil particles, leading to better bonding of soil particles and an improvement of the final strength of soils [12]. The other criticizes the formation of these expansive materials, which may compromise the strength. Needless to say, both of these points of view depend on the generated expansive material, conditions, and sulfate availability in the soil.

**Figure 1:**
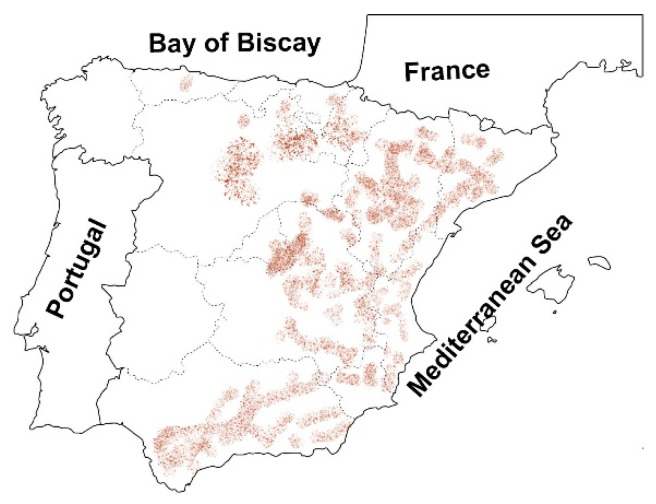
Native gypsum bearing soils in Spain (adapted from [17]).

One of the sources of sulfate is the soil itself. Soils with some amount of sulfate are quite common all around the world [13]. Gypsum, commonly composed of calcium sulfate dihydrate, is a primary source of sulfate in soils and can be found in gypsiferous soils. As reported by Verheye, gypsiferous soils cover approximately 1 million km^2^ of the world’s surface [14]. Gypsiferous soils can be found in countries in the Middle East (e.g., Iraq, Syria, and Iran) and in Europe (especially in Spain), as well as some parts of North Africa and the USA [15]. According to Jara [16], 7.2% of Spain is covered with gypsiferous soil, mainly located in the eastern part of the country as shown in Figure 1.

As mentioned before, the existence of sulfate in soil can be beneficial or problematic due to its reaction with the binder. When lime is added to the soil, cation exchange and flocculation/agglomeration take place almost instantaneously, increasing the pH to around 12. This high pH makes the solution a suitable environment for alumina, silica, and other minerals to react with lime, thereby developing the silica gel (C–S–H) and alumina gel (C–A–H) [3]. The reaction of producing C–S–H and C–A–H are as follows:

(1) $$\mathrm{CaO}+H_{2}O\;\to \mathrm{Ca}{\left(\mathrm{OH}\right)}_{2}$$

(2) $$\mathrm{Ca}{\left(\mathrm{OH}\right)}_{2}\to {\mathrm{Ca}}^{2+}+2{\left[\mathrm{OH}\right]}^{-}$$

(3) $${\mathrm{Ca}}^{2+}+2{\left[\mathrm{OH}\right]}^{-}+{\mathrm{SiO}}_{2}\to C-S-H$$

(4) $${\mathrm{Ca}}^{2+}+2{\left[\mathrm{OH}\right]}^{-}+{\mathrm{Al}}_{2}O_{3}\to C-A-H$$

However, two phenomena may occur when the soil or underground water contains some amount of sulfate. The sulfate may react with alumina and form calcium aluminum sulfate hydrates, eventually leading to the formation of ettringite. Undoubtedly, certain parameters must be met in order for ettringite to form, such as a high temperature, a pH above 10, and enough water. All requirements are satisfied when gypsiferous soil is stabilized with cement or lime, except for water, which may come from underground water or rainwater. It should be pointed out that if the pH drops below 10, ettringite formation stops [18]. The ettringite formation process was proposed by Harris et al. [18] as follows:

(5) $$\mathrm{Ca}{\left(\mathrm{OH}\right)}_{2}\to {\mathrm{Ca}}^{2+}+2{\left(\mathrm{OH}\right)}^{-}\;(\mathrm{Ionization}\;\mathrm{of}\;\mathrm{lime};\;\mathrm{pH}\;\mathrm{rises}\;\mathrm{to}\;12.3)$$

(6) $$\begin{array}{l} {\mathrm{Al}}_{4}{\mathrm{Si}}_{4}O_{10}{\left(\mathrm{OH}\right)}_{8}+4{\left(\mathrm{OH}\right)}^{-}+10H_{2}O\to 4\mathrm{Al}{\left(\mathrm{OH}\right)}^{4-}+4H_{4}{\mathrm{SiO}}_{4}\\ \left(\mathrm{Dissolution}\;\mathrm{of}\;\mathrm{kaolinite}\;\mathrm{at}\;\mathrm{pH}\;>\;10.5\right) \end{array}$$

(7) $${\mathrm{CaSO}}_{4}\cdot 2H_{2}O\to {\mathrm{Ca}}^{2+}+{\mathrm{SO}}_{4}^{2-}+2H_{2}O\;(\mathrm{Dissolution}\;\mathrm{of}\;\mathrm{gypsum})$$

(8) $$6{\mathrm{Ca}}^{2+}+2\mathrm{Al}{\left(\mathrm{OH}\right)}^{4-}+4{\left(\mathrm{OH}\right)}^{-}+3{\left({\mathrm{SO}}_{4}\right)}^{2-}+26H_{2}O\to {\mathrm{Ca}}_{6}{\left[\mathrm{Al}{\left(\mathrm{OH}\right)}_{6}\right]}_{2}\cdot {\left({\mathrm{SO}}_{4}\right)}^{3}\cdot 26H_{2}O$$

The second phenomenon involves the formation of thaumasite. The sulfate may react with calcium silicate hydrate gel in the system and form thaumasite. The rate of reaction can increase at temperatures below 15 °C. Although ettringite and thaumasite have a similar structural arrangement, the expansive capability of thaumasite is less than that of ettringite as it occupies 45% less volume [19]. Moreover, it was reported that ettringite could expand to as much as two times its original size [20], by 250% [21], or by 137% as calculated using molar volume [19].

In cement hydration, it is believed that this expansion leads to better strength if it happens at an early age. However, if the expansion happens at a later age (delayed ettringite formation), some problems can occur in the structure [22]. It has been reported that the soil expansion does not follow the same rate as Portland cement concrete [19]. The authors of [23] found no significant swelling at the early age. They believed this to be due to the void spaces within the stabilized soil, suggesting greater effects for coarse-grained soil [4]. At a later age, the void spaces inside the soil are filled up, resulting in a more rigid product with fewer pore spaces. Nevertheless, as the ettringite swells and no more pore spaces are left to fill, the swelling pressure is applied to other parts of the structure and can lead to potential catastrophe [23]. One example was reported by Chen et al. [24], where an 8.8 mile section of a road in Texas, USA, which was treated with lime, was damaged and caused 12.7 million USD worth of damage.

Nevertheless, ettringite formation does not only depend on sulfate content [19,25], and is not always expansive. It depends on many factors such as composition, curing period time and temperature, water availability, reactive phase availability [23,25], compatibility with other cement phases [9], and amount of lime. Studies have reported that the swelling potential of sulfate-rich soils is decreased when they are treated with low C_3_A binders such as ground granulated blast furnace slag (GGBS) [26]. Seco et al. [27] found that stabilization with a byproduct from the calcination of natural MgCO_3_ rocks (defined as PC-8) could significantly decrease swelling while maintaining a similar or better strength compared to stabilization with lime. Eyo et al. [28] conducted a study using RoadCem (RC), an additive for nanotechnology manufacturing. It was concluded that using 1% RC and replacing cement with GGBS could decrease the swelling. Fly ash geopolymer has also been shown to be a viable solution by increasing pozzolanic reactions [29]. However, it requires supplemental additives. The use of fly ash, particularly low-calcium fly ash, can reduce the rate of heat evolution and the magnitude of the temperature rise in concrete, especially at high replacement levels. Another way to reduce swelling, at least for soil treated with lime, is mellowing [18,30]. It has been shown that mellowing can significantly decrease swelling and double the sulfate content.

Another important factor influencing the structure and strength of stabilized soils in cold regions is represented by freeze–thaw cycles [31]. Yan et al. [32] investigated the characteristics of unconfined compressive strength and pore distribution of lime–fly ash loess mixtures under freeze–thaw cycles and drying–wetting cycles through a series of experiments in the laboratory. The authors showed that the freeze–thaw cycles caused frequent phase changes and water transference in samples, which continuously lowered the friction and bite forces between the soil particles, eventually leading to lower strength.

Waste paper ash (WPA), a byproduct of recycling paper, varies in terms of its chemical and physical properties, generally depending on the raw material used during incineration. However, in most cases, WPA contains cementitious properties [33,34] and, to some extent, follows the same pattern as cement. After mixing WPA with water, lime makes the solution alkaline (around 12) [35].

In the previous study, the usability of WPFA as a binder was discussed [35]. In summary, WPFA was successfully used as the sole binder to stabilize the given soil. However, the durability of WPFA in the presence of a sulfate source was not considered. The study of the durability of WPFA is essential because of the similarities between WPFA and cement. Similarly to cement, WPFA in the presence of sulfate could swell and eventually lead to structural damage.

Moreover, swelling in soils by the formation of ettringite depends on many factors such as temperature, water and sulfate content, and time [23,25]. Therefore, this paper studies the long-term effect of soil stabilized using WPFA in the presence of different sulfate concentrations, by means of measuring the mechanical performance and swelling. The study valued mineralogical changes using XRD, thermogravimetry analysis (TGA), Fourier-transform infrared spectroscopy (FTIR), and scanning electron microscopy (SEM) analyses. For this purpose, all tests were conducted in different conditions (at 5 °C and 20 °C with different sulfate solutions), and the results were compared with a commonly used binder (CEM IV). This study’s findings will further reveal the usability of WPFA as a binder even in a harsh environment and would be a major importance in assessing WPFA in comparison with traditional cement.

## 2. Materials and Methods

### 2.1. Soil, Stabilizers, and Reagent

**Figure 2:**
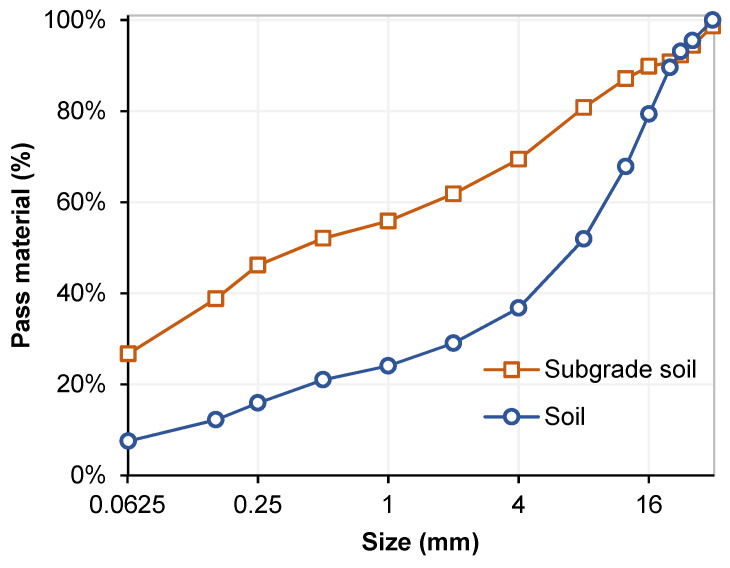
Particle size distribution of soils.

The treated soil was collected from the suburbs of the city of Zaragoza, Spain (north of Spain), where the metropolitan area is predominantly composed of soils contaminated with sulfate. Given the low load-bearing capacity of these soils due to their physical–mechanical properties, they are not used in construction work; thus, the stabilization of soils with cement materials is a practical solution. The goal was to stabilize a 30 cm layer of this soil. Upon further inspection, the subgrade soil (below the treated soil, around 0.5 m depth) showed a high amount of sulfate concentration (1.4% according to EN 103201). Hence, the study also considered the underlying soil. The properties of tested soils such as particle size distribution, liquid limit, plastic limit, sulfate content, and pH values are shown in Figure 2 and Table 1.

The stabilizers included a pozzolanic Portland cement (CEM IV B(Q) 32.5 N) and WPFA. The cement consisted of a pozzolanic cement with additional calcined natural pozzolana (Q) and a resistance class of 32.5 N; and the waste paper fly ash was derived from paper manufacturing. The WPFA studied in this study was supplied by Saica (Sociedad Anonima Industrias Celulosa Aragonesa), a Spanish pulp and paper manufacturer that uses only recycled paper as raw material.

The chemical composition of all raw materials (both soils and stabilizers) was determined by X-ray fluorescence, using a Philips/PANalytical spectrometer, model PW2400. The main elements in both soils were calcium and silicon. The main elements in WPFA and cement were calcium, silicon, and aluminum. There were some traces of magnesium and chlorine in WPFA, as shown in Table 2.

**Figure 3:**
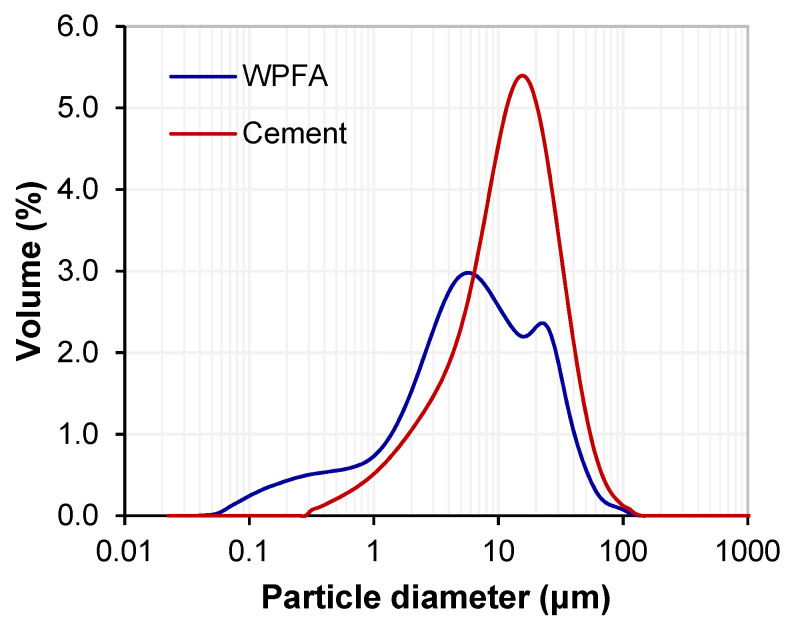
Particle size distribution of WPFA and WPBA.

Figure 3 shows the particle size distribution of PC and WPFA. The as-received WPFA contained particles with a d_50_ of ~6.4 μm, whereas the cement contained particles with a d_50_ of 11.8 μm, showing a far coarser particle size than WPFA.

This study applied the powder diffraction technique to identify the crystalline phases in soils and stabilizers using a Philips X-ray diffractometer with a PANalytical X’Pert PRO MPD Alpha 1 diffractometer using Cu Kα radiation (λ = 1.5406 Å, 45 kV–40 mA). The results were interpreted using EVA software (database PDF-2).

**Figure 4:**
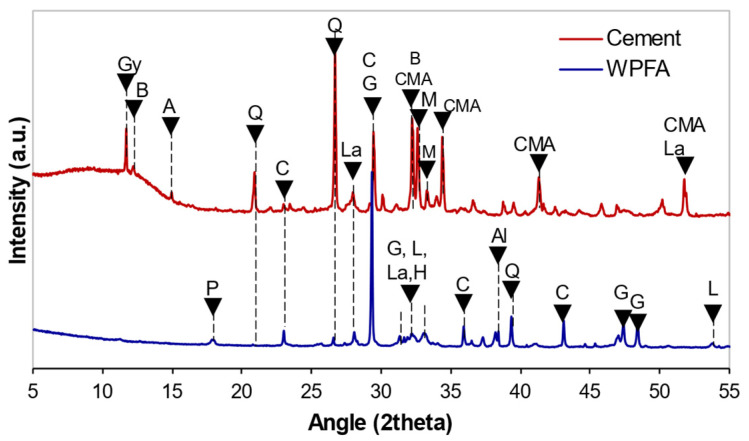
Diffractograms of WPFA and cement. Gy: gypsum, B: brownmillerite, A: albite, Q: quartz, La: larnite, M: mayenite, P: portlandite, C: calcite, L: lime, G: gehlenite, H: halite, CMA: calcium magnesium aluminum oxide silicate, A: aluminum.

**Figure 5:**
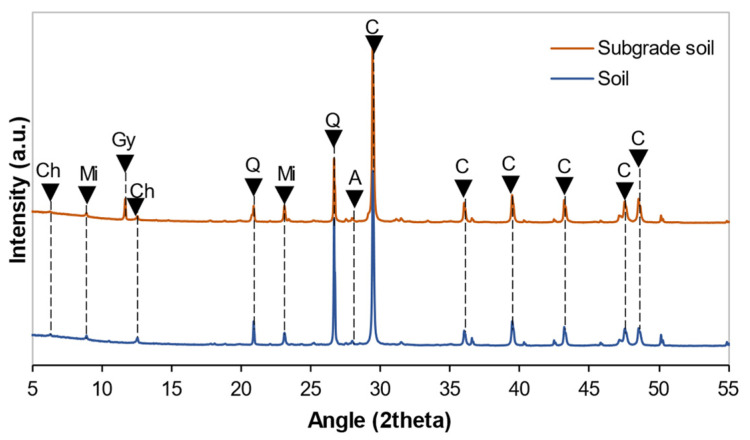
Diffractograms of soil and subgrade soil. Gy: gypsum, A: albite, Ch: chamosite, Q: quartz, Mi: mica, C: calcite.

The presence of calcite, lime, quartz, larnite, aluminum, and halite was recorded in WPFA, as shown in Figure 4. Moreover, a tiny amount of portlandite could be found due to moisture in the environment. The minerals presented in cement were quartz, calcite, mayenite, brownmillerite, gypsum, tricalcium aluminate, larnite, and calcium magnesium aluminum oxide silicate. The soil and subgrade soil were composed of quartz, calcite, albite, biotite, chamosite and mica, as shown in Figure 5.

Moreover, in addition to these materials, calcium sulfate 2-hydrate (CaSO_4_·2H_2_O) from Panreac was used as a reagent. It was mixed with water in order to facilitate sulfate attack of the test specimens. The amount of calcium sulfate is described in Section 2.3.

### 2.2. Sample Preparation

The studied soil was part of an experimental trial, located in Villamayor de Gállego, a small village near Zaragoza, Spain. The goal was to treat the soil for heavy traffic usage, whereby a minimum of 2.5 MPa according to the unconfined strength test (UCS) was required at 7 days in this case. Table 3 shows the test design parameters such as the binder content and UCS for both soil + WPFA and soil + cement.

### 2.3. Procedure for Measuring Swelling

An in-house experiment was designed to characterize the effect of swelling under sulfate attack on stabilized soils in the long term. The experiment measured one-dimensional swelling/shrinkage in the vertical direction of a confined specimen. This allows more flexibility and a greater experimental duration without damaging the sample. PVC molds were fabricated a thickness of 0.5 cm, height of 20 cm, and diameter of 10 cm. As the base, perforated PVC was also used, with a thickness of 0.5 cm. The base and the mold were glued together using eight screws.

**Figure 6:**
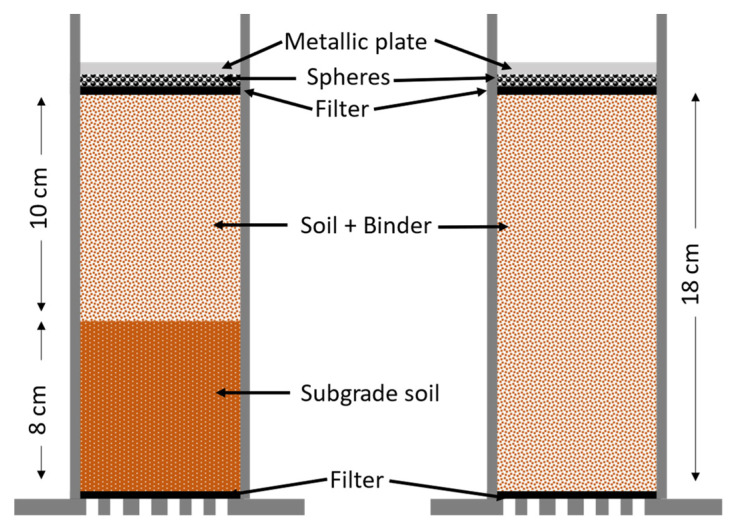
Schematic of swelling sample.

The preparation of soil consists of grinding the soil to obtain a maximum particle size of 16 mm. The soil was weighed and mixed with binder (either 5 wt.% WPFA or 3 wt.% cement) and water according to Table 3. After mixing thoroughly, the mix was then poured into the mold in five layers and compacted. For samples with WPFA, after mixing with water, a 30 min delay was considered before pouring and compacting. This delay time allowed the WPFA to gain better workability and performance and as well as reduced swelling to some extent. After fabricating the samples, a 125 g sphere was added to level the top layer of the soil. Finally, to facilitate measurements, a metallic plate was added to the top of the samples. For clarity, a schematic of the mold is shown in Figure 6.

After fabrication, the samples were cured for 7 days. In total, 36 samples were fabricated. Two temperatures were considered (5 °C and 20 °C). To determine the effect of sulfate on the samples, three sulfate solutions were considered. In the first (W1), only tap water was used. For the second batch (W2), the samples were fabricated using subgrade soil to determine the effect of underlying soil on the stabilized soil. Additionally, to avoid any loss of sulfate concentration from subgrade soil to the water bath, 2.5 g/L calcium sulfate was added to the water bath. Lastly, for the third batch (W3), 20 g/L calcium sulfate was added to the water bath. Each experiment for WPFA (in terms of water batch and temperature) was conducted in quadruplicate, whereas experiments with cement were conducted in duplicate. Due to the lack of research and the possible greater heterogeneity on the behavior of WPFA exposed to sulfate concentrations, more samples were manufactured. Table 4 shows the number of samples and the wetting/drying conditions for each sample batch.

The wetting/drying cycles were carried out immediately after day 7. The samples were placed inside a designated water bath for 1 week, and 2 weeks to dry. This cycle was repeated for 800 days. After each cycle, the weight and the swelling were measured.

A precise displacement device was used to measure the swelling/shrinkage in the soil samples as described in previous work [36].

**Figure 7:**
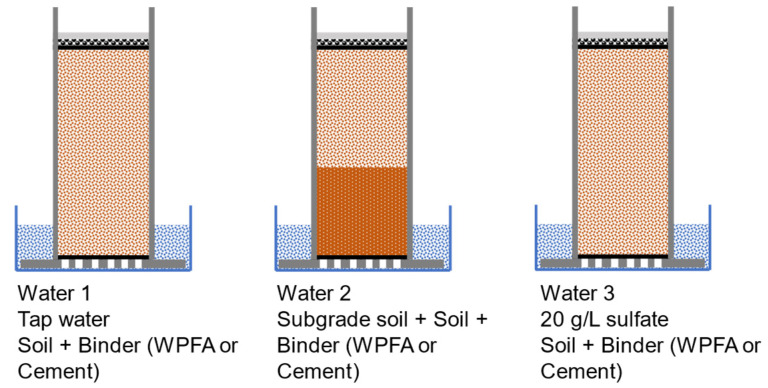
Water bath and different sulfate solutions.

Samples were placed in a water bath at 20 °C, and 90 ± 5% humidity, or at 5 °C. The water level was maintained around 1.5 ± 0.5 cm above the samples to allow the water to be drawn up into the sample via capillary action. Figure 7 shows a schematic view of wetting portion with different sulfate solutions.

### 2.4. Microstructural Studies of Stabilized Soil

To study the behavior of the soil stabilized with binders, 100 g of soil was ground to a particle size of 63 µm. Later, it was mixed with 5% WPFA or 3% cement and the three different solutions mentioned in the previous section (W1: tap water, W2: containing underlying soil, W3: containing 20 g/L sulfate). After mixing thoroughly, it was poured into a container and left to cure at two different temperatures (5 and 20 °C). This experiment was conducted to accelerate the hydration process and promote the appearance of the other phases such as ettringite or thaumasite.

The hydration of the samples was stopped at different curing ages (30, 180, and 360 days) using the solvent exchange method. Then, the samples were pulverized for characterization by XRD, thermogravimetry analysis (TGA), scanning electron microscopy (SEM), and FTIR. For TGA, a Mettler Toledo model TGA/DSC 1 Thermal Analyzer was used with 10 µg of material at temperatures within the interval 30–1000 °C, N_2_ flow of 50 mL/min, and a heating rate of 10 °C/min. Before each test, the samples were stabilized at 100 °C for 15 min. A Frontier FTIR spectrometer (Perkin Elmer) was used to acquire 16 scans with a spectral resolution of 4 cm^−1^ over a range of 4000–400 in attenuated total reflection (ATR) mode.

Scanning electronic microscopy (SEM) images of soil stabilized with WPFA or cement at 360 days were obtained to verify the formation of ettringite, using a FEI scanning electronic microscope equipped with an energy-dispersive X-ray microscopy device model ESEM Quanta 200, XTE 325/D8395.

### 2.5. Unconfined Compressive Strength

To evaluate the changes in strength of the stabilized soil under sulfate attack, a UCS test was carried out. The samples were prepared in accordance with EN13286 and cured for 7 days at 90 ± 5% RH. Then, the samples were placed inside a tray, and the water solution (W1, W2 or W3) was poured into the tray. For this test, the samples were exposed to the solution for 1 week, and then left to dry at room temperature for 2 weeks. The experiments were carried out over a 1 year period, and then the compressive strength test was performed.

## 3. Results and Discussion

### 3.1. Unconfined Compressive Strength

**Figure 8:**
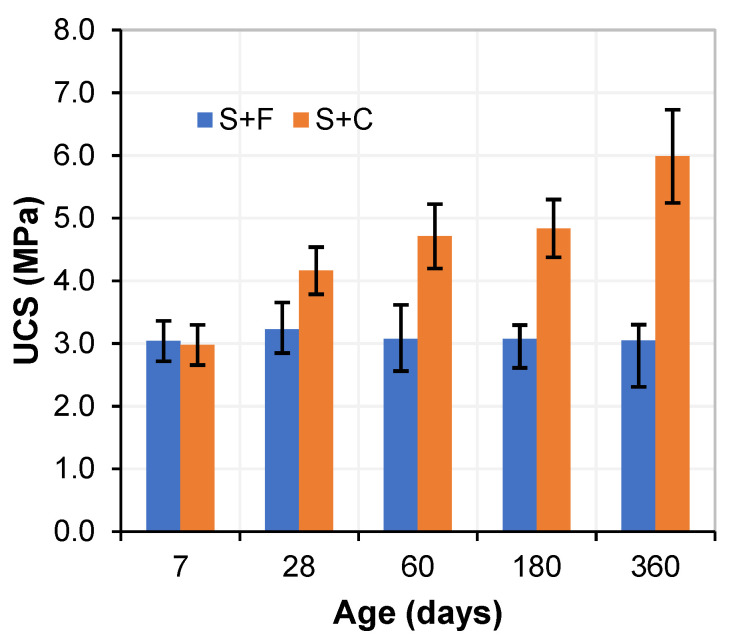
UCS for soil stabilized with WPFA or cement.

Figure 8 shows the results of the unconfined compressive strength (UCS) test cured at different ages in an optimum environment (90 ± 5% RH, and 20 °C) for soil stabilized with WPFA or cement. In the case of soil treated with WPFA, the UCS tended to stay nearly constant, obtaining the maximum value over a short period of time (3 MPa at 7 days) compared to cement samples. Moreover, as the cement was pozzolanic, its strength doubled over the 1 year period.

**Figure 9:**
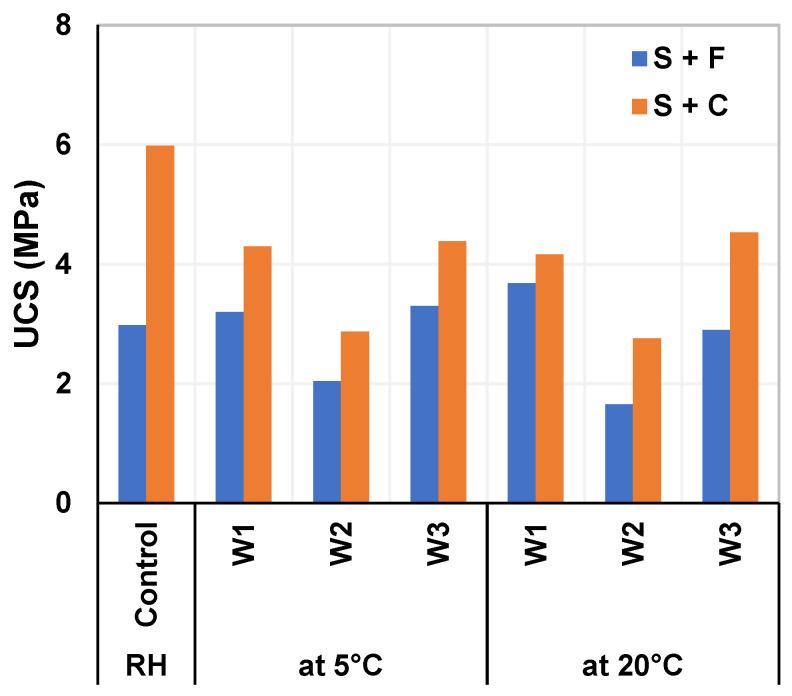
UCS at 360 days for soil stabilized with different binders, temperatures, and sulfate solutions.

Figure 9 shows the results of the unconfined compressive strength (UCS) test for soil stabilized with WPFA or cement at 360 days under different conditions. The first part shows the samples cured in a humid room (at 20 °C, with 90 ± 5% humidity), while the second and third parts show samples subjected to the above-described wetting/drying cycles for 1 year.

At first glance, the strength of the WPFA samples remained fairly constant across all conditions (~3 MPa) for W1 and W3. This is due to the fact that WPFA gained most of its strength in the first 7 days of curing as shown in Figure 8. However, for samples exposed to W2, a decrease of 31 to 45% was observed. This decrease can be justified with the fact that only a portion of the sample (only 10 cm) was stabilized, and no treatment was applied to the subgrade soil.

In the case of cement samples, a significant decrease from 5.98 MPa to ~4.3 MPa (a decrease of 28%) was observed following the wetting and drying cycles. Furthermore, this decrease remained unaffected by the various sulfate concentrations. Additionally, the strength of cement samples exposed to W2 decreased, similarly to WPFA samples, because only 10 cm of this sample was treated.

There are two possible explanations for the decrease in strength in cement samples. The cement used in this study was pozzolanic and was, thus, characterized by lower strength gains at an early age. The cement samples reached 50% strength after 7 days compared to that after 360 days. Moreover, since the samples were subjected to wetting/drying cycles, during the drying period, the cement did not completely develop in terms of hydration reactions and strength. Likewise, due to these wetting/drying cycles, internal cracks may have been produced inside the material, which would have affected the final resistance.

### 3.2. Swelling in Treated Soil

The average confined swellings and weight changes at 800 days are shown in Table 5. The swelling of samples showed no significant changes during the 800 day period. The greatest change was recorded for the soil stabilized with WPFA and tap water at 20 °C with a value of 0.15%, showing a small amount of shrinkage in the sample. For cement samples, the greatest change was recorded to the soil stabilized with cement and water with 20 g/L sulfate content at 20 °C with a value of −0.11%. Both WPFA and cement samples exhibited a small amount of shrinkage. Moreover, samples at 20 °C showed more shrinkage than those at 5 °C. Nevertheless, these values were too small to affect the properties of the samples.

**Figure 10:**
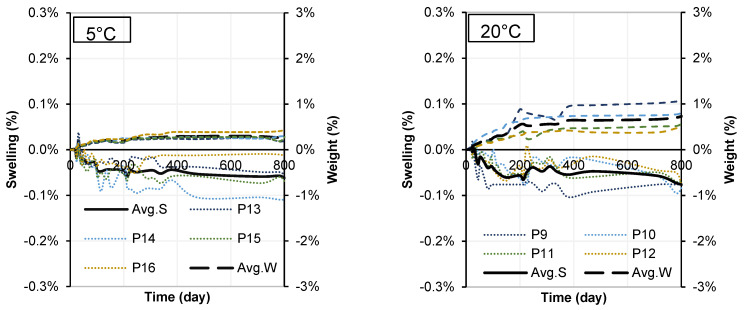
Swelling results in Sg + S + F + W2 at different temperatures.

**Figure 11:**
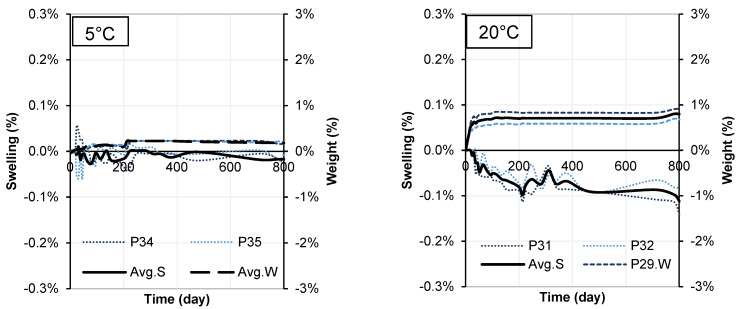
Swelling results in Sg + S + C + W2 at different temperatures.

Another point is that the weight of the samples was also increased following the drying/wetting cycles. The maximum weight changes were recorded for the soil stabilized with WPFA and W3 at 20 °C (1.96%) and the soil stabilized with cement + W1 at 20 °C. Figure 10 and Figure 11 show the changes in each sample (SG + S + F + W2 and SG + S + C + W2) over the 800 days, as well as their average weight. For the sake of saving space, the remaining figures are presented in Appendix A.1.

### 3.3. Microstructural Studies of Treated Soil

**Figure 12:**
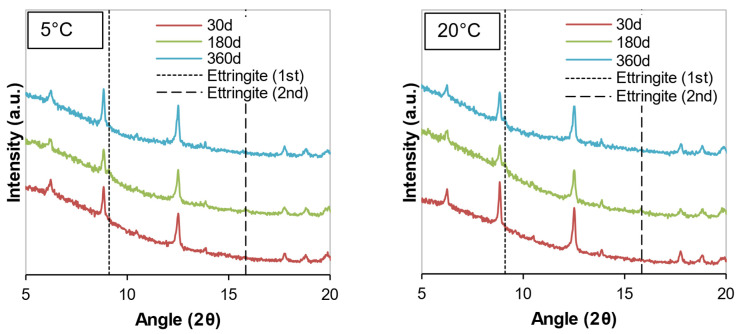
Diffractograms of S + C + W1 at different temperatures.

**Figure 13:**
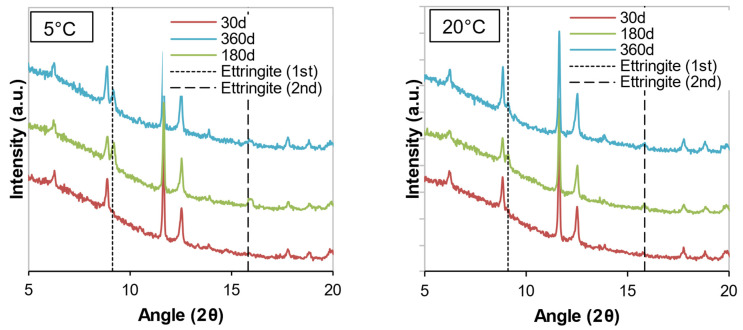
Diffractograms of Sg + S + C + W2 at different temperatures.

**Figure 14:**
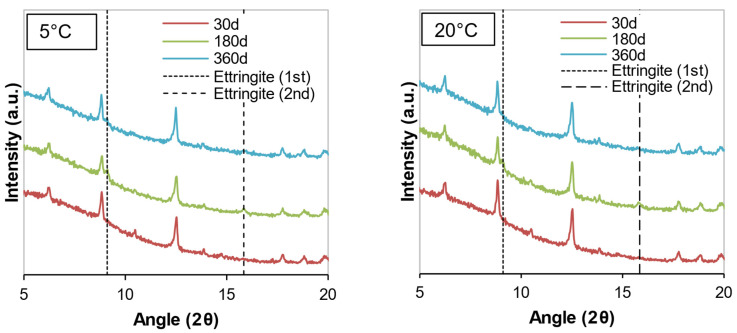
Diffractograms of S + C + W3 at different temperatures.

**Figure 15:**
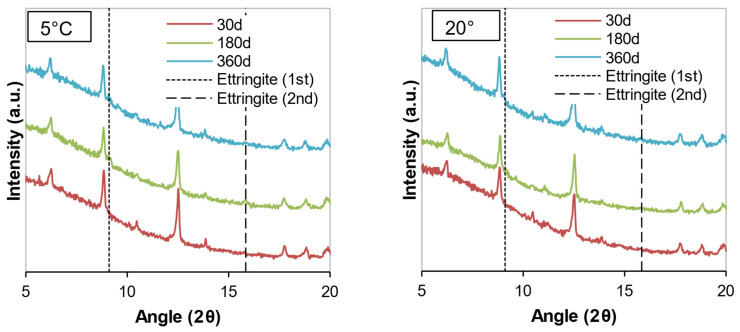
Diffractograms of S + F + W1 at different temperatures.

**Figure 16:**
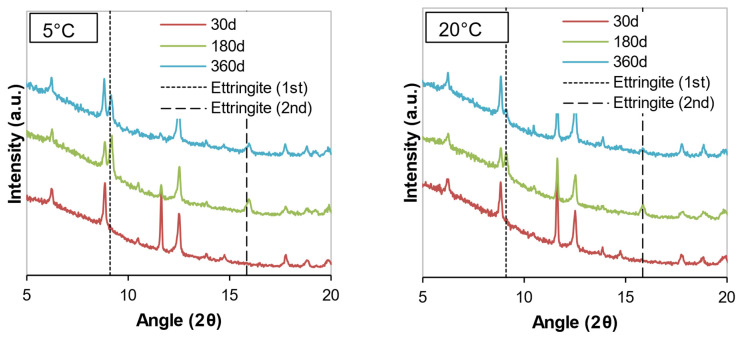
Diffractograms of Sg + S + F + W2 at different temperatures.

**Figure 17:**
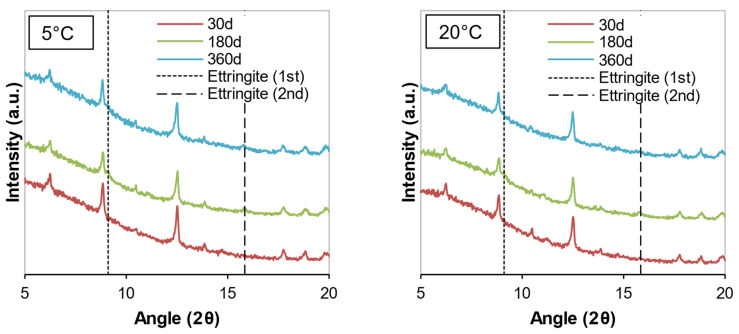
Diffractograms of S + F + W3 at different temperatures.

The mineralogical compositions of the soil samples stabilized with cement or WPFA for 30, 180, and 360 days are shown in Figure 12, Figure 13, Figure 14, Figure 15, Figure 16 and Figure 17. Most of the peaks identified were similar in all the cases, corresponding to soil and subgrade soil, and no significant changes were observed during the 1 year period. For the sake of clarity, only a portion of each figure (from 5°–20° 2θ) is shown to observe the evolution of AFt–AFm and gypsum in the system.

For samples stabilized with W1 and W3, very little ettringite was formed, and the maximum peak was reached after 180 days. However, at 360 days, the amount of ettringite decreased for both WPFA and cement samples. For samples containing subgrade soil, the gypsum peak at 11.61° (2θ) was lowered, indicating the consumption of gypsum, while the formation of ettringite increased to its maximum peak after 180 days. At 360 days, there were still some traces of gypsum in the system, and the ettringite peaks were reduced. The variation in temperature did not play an essential role in the formation of thaumasite or other phases.

**Figure 18:**
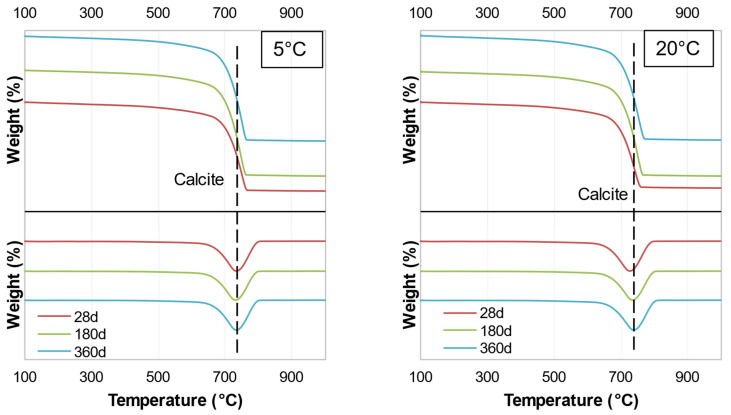
TGA traces of Sg + S + F + W2.

**Figure 19:**
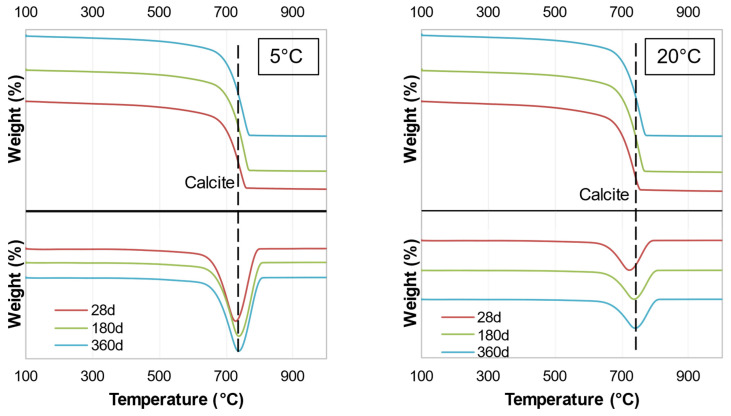
TGA traces of Sg + S + C + W2.

TGA analysis was conducted to compare the results of the samples with different hydration ages, different sulfate concentrations, and different temperatures. The major peak was related to calcite at 731 °C, originating from the soil and binder, which remained the same throughout the hydration. Unfortunately, the TGA results did not provide an accurate measure of phases other than calcite. Due to space limitations, only one figure is presented for each binder (Figure 18 and Figure 19), i.e., subgrade soil and soil with each binder at temperatures of 5 and 20 °C; the remaining figures are presented in Appendix A.2. The results indicated that there were no major changes as a function of hydration age or sulfate concentration. These results also indicated that the amount of binder was insufficient (5 wt.% WPFA or 3 wt.% cement) to affect the system significantly.

**Figure 20:**
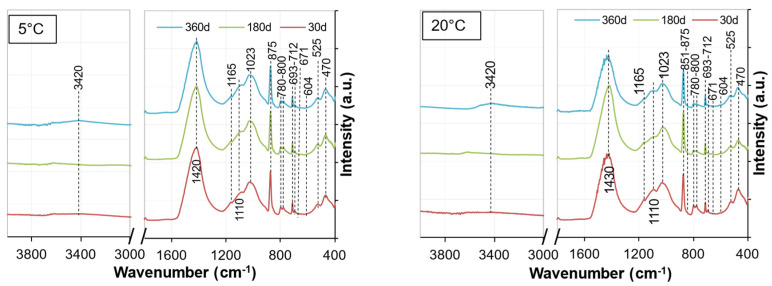
FTIR spectra of Sg + S + FA + W2.

**Figure 21:**
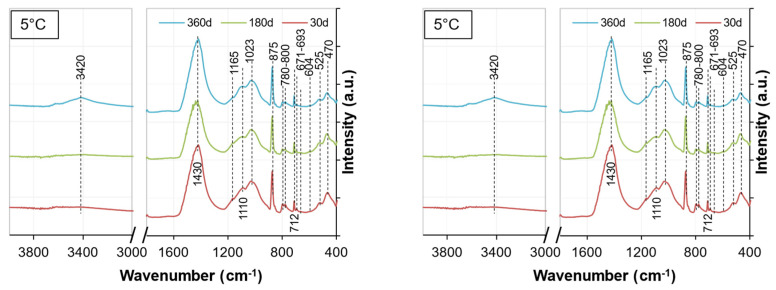
FTIR spectra of Sg + S + C + W2.

Figure 20 and Figure 21 show the FTIR spectra for the soil stabilized with WPFA or cement at curing ages of 30, 180, and 360 days. Table 6 provides the analyses of FTIR spectra for the soil stabilized with WPFA or cement. The similarities in the FTIR spectra confirmed the quite similar reaction products formed for both cement and WPFA. The absorption bands at 712 cm^−1^, with a narrow band around 875 cm^−1^ and a strong band at 1420 cm^−1^, were assigned to the asymmetric stretching vibration of C–O bonds of calcite. The absorption bands at 470 and 525 cm^−1^ could be assigned to the bending vibration of Si–O [37]. Moreover, the small peaks at 604 and 671 cm^−1^ were assigned to the stretching and bending modes of sulfate. The two small bands at 780 and 800 cm^−1^ could be assigned to vibration of Al–O. The peaks within the range of 1060 to 1165 cm^−1^ were due to the vibration of SO4. Due to the similarities and overlapping peaks between the gypsum and AFm phases, distinguishing them in the system was complex. It is believed that the amount of gypsum in the system was high enough to consume all reactive aluminum phases and still be present in the XRD and FTIR spectra after 360 days for W2. The spectra for W1 and W3 are presented in Appendix A.3.

**Figure 22:**
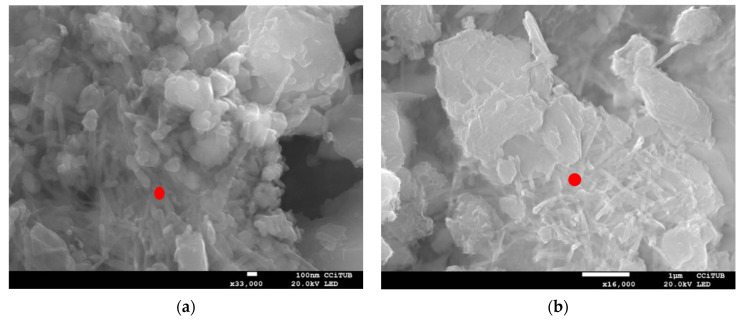
SEM images of soils treated with WPFA (**a**) and cement (**b**) at 360 days. Red dots indicate the formation of ettringite.

To verify these findings, the samples were observed by SEM after 360 days. In both cases, small traces of ettringite could be observed, as shown by red dots in Figure 22.

## 4. Conclusions

A 2 year comparison study was conducted to verify the usability and durability of waste paper fly ash and cement IV as binders in soil samples in different sulfate concentrations. The preliminary results indicated that up to 5% WPFA and 3% cement IV are well suited for a harsh sulfate environment. Acceptable performance is also expected to be obtained with lower sulfate content than proposed in this study. Moreover, future work on determining the feasibility of using WPFA to stabilize different types of soil, with higher sulfate contents should be carried out.

The results of this study revealed the following:

- The unconfined compressive strength (UCS) results after 360 days of WPFA treated soil with different sulfate solutions and different temperatures showed no major changes. The UCS for WPFA after wetting/drying cycles stayed at around 3 MPa for W1 and W3. However, for W2, UCS decreased to about 1.7 MPa, a decrease of 56%, which was expected since only a portion of samples was stabilized with WPFA.
- The UCS of cement samples was significantly reduced compared to those cured at the optimum humidity (90% ± 5% relative humidity) from 6 MPa to 4.3 MPa for both W1 and W3. For samples with W2, the UCS is also decreased by around 2.9 MPa. Although the cement strength was lowered, it was still high enough to surpass the minimum requirement of 2.5 MPa mentioned by Spanish roads and bridges standards.
- The swelling in the samples was tested over 2 years while being exposed to different sulfate solutions and temperatures. The different temperatures and sulfate concentrations had no significant effect on the swelling in soils treated with WPFA or cement. In most cases, a minor shrinkage of around 0.1% was observed. Meanwhile, the weight of the samples increased slightly between 0.02% to 2%.
- Furthermore, the microstructural studies revealed that the formation of ettringite reached its peak after 180 days for samples in contact with subgrade soil. This formation was highest with subgrade soil (W2), intermediate for W3 (20 g/SO_4_), and lowest for W1 (tap water). Moreover, after 360 days, the ettringite was partially converted into other poor crystalline AFm phases (e.g., AFm–CO_3_, AFm–SO_4_, or Friedel’s salt), which were not detected due to complexity in the system.

Finally, as shown in this work, extensive laboratory research in the field of waste utilization in civil engineering is necessary for a better understanding of their use and feasibility. This work demonstrated that it is possible to use WPFA as the sole binder for soil stabilization, even in a harsh environment, replacing the commonly used cement.

## Figures and Tables

**Table 1:** Properties of the tested soils.

Test Description	Test Standard	Test Result	
		Soil	Subgrade Soil
USCS soil classification	ASTM D2487	GP–GM	SP–SM
Liquid limit (%)	UNE 103103	Non plastic	Non plastic
Plasticity index	UNE 103104	Non plastic	Non plastic
Free swelling	UNE 103601	No swelling	No swelling
Organic matter	UNE 103204	0.87%	1.0%
Soluble sulfate	UNE 103201	0.27%	1.4%
Optimum moisture (%)	UNE 103501	8.2%	7%
pH	EN-12457-2	11.8	8.11

**Table 2:** Chemical composition of raw materials.

Chemical Composition	Mass Fraction (%)			
	WPFA	Cement IV	Soil	Subgrade Soil
CaO	48.86	35.33	35.3	38.74
SiO_2_	12.58	38.11	27.52	18.34
Al_2_O_3_	12.55	10.9	3.75	3.23
MgO	1.82	1.51	0.97	0.88
Fe_2_O_3_	1.01	5.59	2.17	1.55
ClO	2.28	-	0.06	0.04
TiO_2_	1.20	0.42	0.21	0.17
SO_3_	0.96	2.61	0.6	2.84
P_2_O_5_	0.8	2.72	1.14	1.10
Other	1.6	-	-	-
LOI	17.8	2.5	28.2	33.1
Free lime content	6.36	-	-	-
Density (g/cm^3^)	2.68	3.00	-	-

**Table 3:** Test design parameters for soil stabilization.

	Soil + WPFA	Soil + Cement
Binder content (wt of soil)	5%	3%
Water content	8.2%	7%
Modified proctor density	1.98 g/cm^3^	2.05 g/cm^3^
UCS at 7 days	3.04 MPa	2.98 MPa
Applied delay before compaction	30 min	-

**Table 4:** Confined swelling samples with different conditions. S: soil, Sg: subgrade soil, F: WPFA, C: cement.

Number of Samples	Samples	Wetting/Drying Condition
4	S + F + W1	20 °C/95% RH
4	S + F + W1	5 °C/dry
4	Sg + S + F + W2	20 °C/95% RH
4	Sg + S + F + W2	5 °C/dry
4	S + F + W3	20 °C/95% RH
4	S + F + W3	5 °C/dry
2	S + C + W1	20 °C/95% RH
2	S + C + W1	5 °C/dry
2	Sg + S + C + W2	20 °C/95% RH
2	Sg + S + C + W2	5 °C/dry
2	S + C + W3	20 °C/95% RH
2	S + C + W3	5 °C/dry

**Table 5:** Average confined swelling and weight changes. S: soil, Sg: subgrade soil, F: WPFA, C: cement.

Specimen Type	Average Swelling Changes	Average Weight Changes
S + F +W1 (5 °C)	−0.14%	1.44%
Sg + S + F + W2 (5 °C)	−0.06%	0.30%
S + F + W3 (5 °C)	−0.03%	1.78%
S + F + W1 (20 °C)	−0.11%	1.84%
Sg + S + F + W2 (20 °C)	−0.10%	0.73%
S + F + W3 (20 °C)	−0.08%	1.96%
S + C + W1 (5 °C)	−0.05%	0.44%
Sg + S + C + W2 (5 °C)	−0.02%	0.19%
S + C + W3 (5 °C)	0.02%	0.02%
S + C + W1 (20 °C)	−0.05%	1.23%
Sg + S + C + W2 (20 °C)	−0.09%	0.40%
S + C + W3 (20 °C)	−0.11%	0.80%

**Table 6:** Interpretation of peak positions observed in FTIR spectra of soil stabilized with WPFA or cement.

Wavenumber (cm^−1^)	Bond	Reference
470	υ_2_ Si–O_4_^4−^	450 [38,39], 455 [40], 465 [41]
525	υ_4_ Si–O_4_^4−^	525 [40], 521 [42]
604	SO_4_	603.72 [13]
671	SO_4_	669.3 [13]
693	Si–O	692 [41]
712	υ_4_ CO_3_	713 [43], 714 [42]
780	Al–O	786 [39]
800	Al–O	814 [39]
851	υ_3_ CO_3_^2−^	849 [42]
875	υ_4_ CO_3_^2−^	875 [40], 876 [42], 874 [44]
1023	Asymmetric stretching Si–O	950–1100 [41]
1060–1165	υ_3_ SO_4_^2−^	1105 [40], 1113 [43], 1116.78 [13], 1116 [22], 1120 [45], 1141.95 [13], 1170 [45]
1420–1430	υ_2_ CO_3_^2−^	1425 [13,40], 1458 [42], 1460 [44], 1429 [46], 1422 [22]
3420	υ_1_ + υ_3_ H_2_O	2700–3600 [44], 3433 [46], 3430 [47], 3425 [22], 3450 [38,40], 3444 [43]

**Figure A1:**
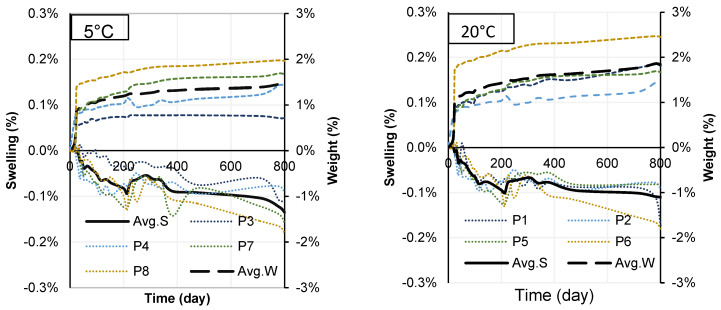
Swelling results in S + F + W1.

**Figure A2:**
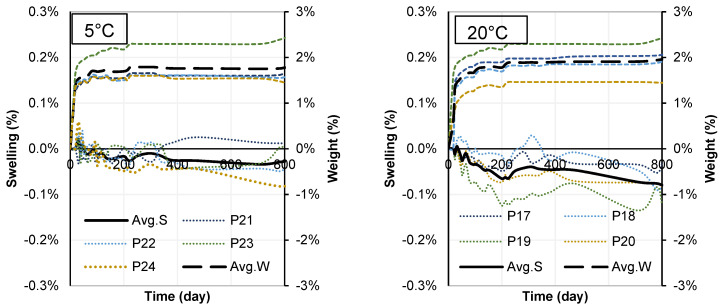
Swelling results in S + F + W3.

**Figure A3:**
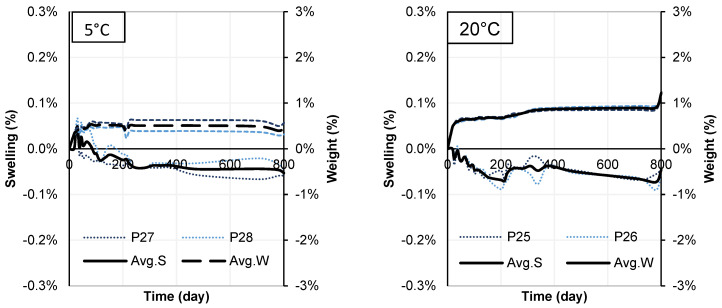
Swelling results in S + C + W1.

**Figure A4:**
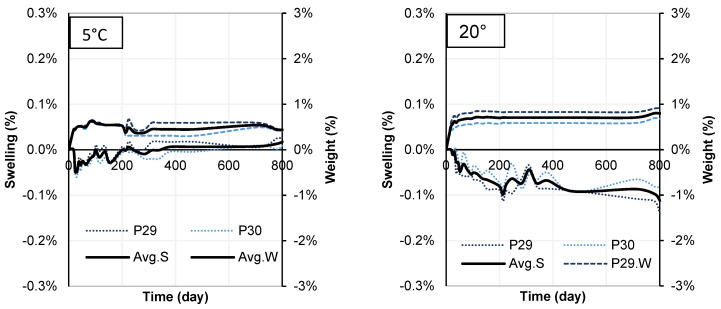
Swelling results in S + C + W3.

**Figure A5:**
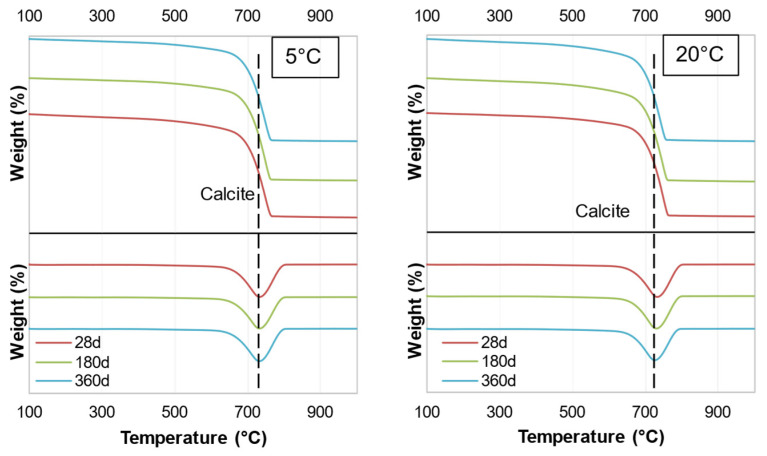
TGA results in S + F + W1.

**Figure A6:**
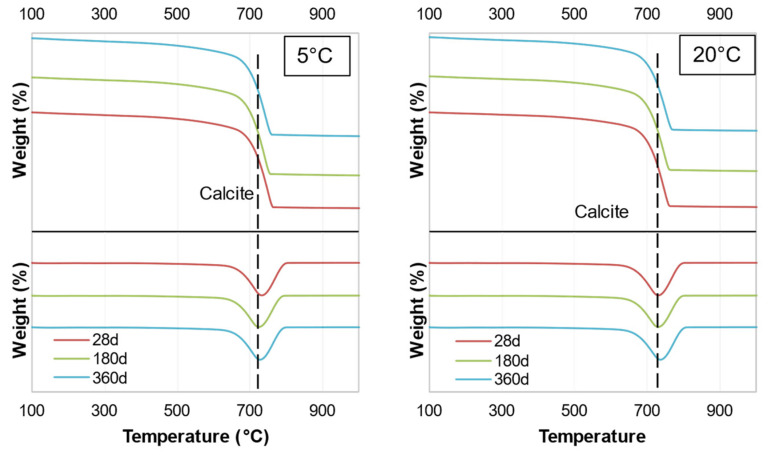
TGA results of S + F + W3.

**Figure A7:**
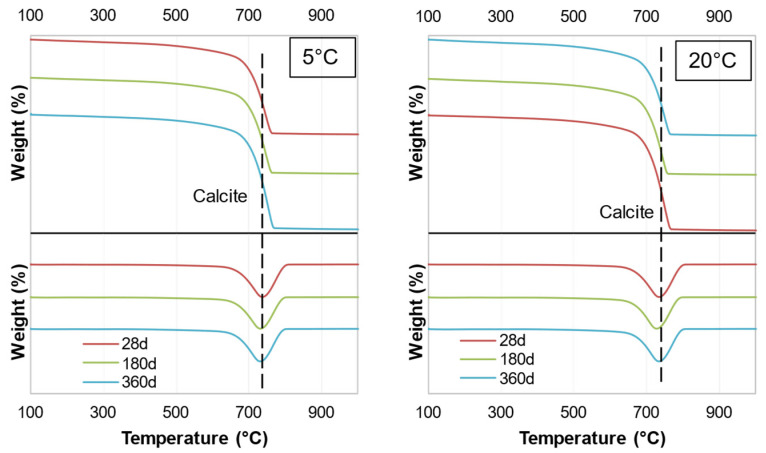
TGA results of S + C + W1.

**Figure A8:**
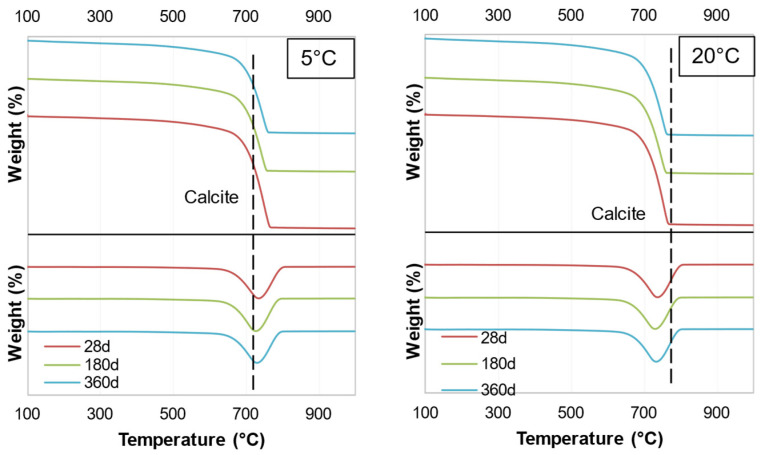
TGA results of S + C + W3.

**Figure A9:**
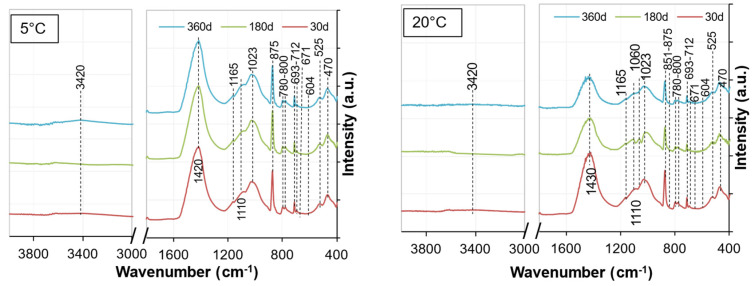
FTIR results of S + F + W1.

**Figure A10:**
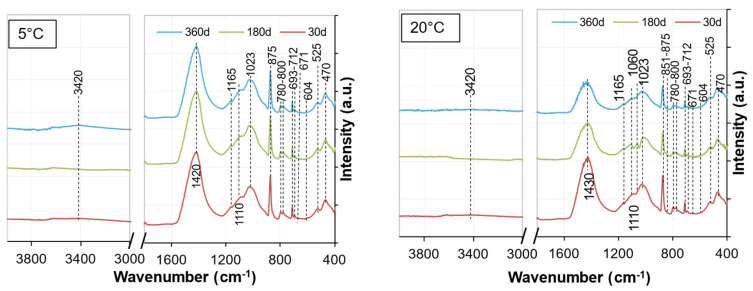
FTIR results of S + F + W3.

**Figure A11:**
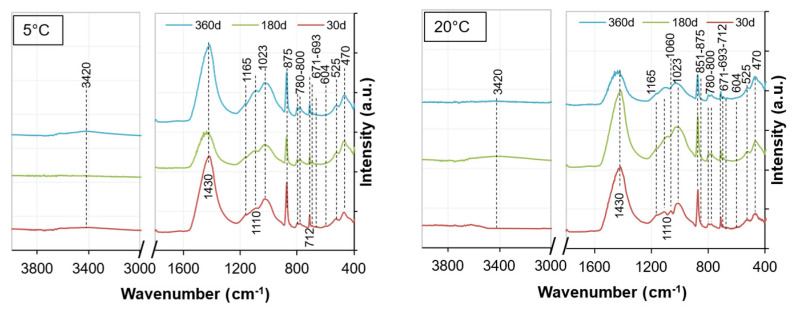
FTIR results of S + C + W1.

**Figure A12:**
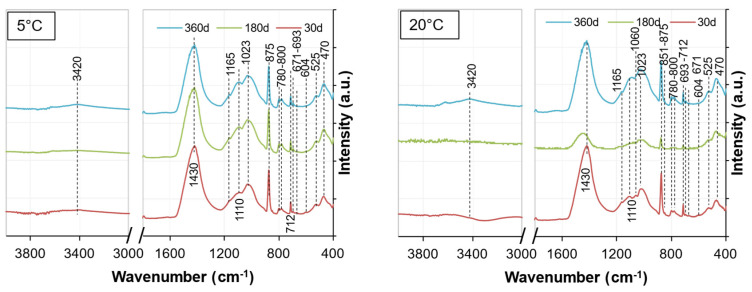
FTIR results of S + C + W3.

## Appendix A

### Appendix A.1. Swelling Results

The complete swelling figures are presented in this section. Figure A1 represents the swelling/shrinkage in soil stabilized with WPFA and tap water. The maximum weight change belongs to samples at 20 °C which is 1.84%. Additionally, no major swelling/shrinkage was observed in the samples exposed to tap water.

Figure A2 shows the swelling/shrinkage in soil stabilized with WPFA and 20 g/L sulfate solution in water (W3). The maximum weight change belongs to samples at 20 °C. The shrinkage in these samples was lower than those exposed with tap water.

The swelling and weight change in soil stabilized with cement and tap water are shown in Figure A3. The weight change in the sample at 20 °C was 1.23%, and the swelling/shrinkage in both temperatures are equal.

Figure A4 presents the soil stabilized with cement and W3. In this also no major changes were observed.

### Appendix A.2. TGA Results

The complete TGA study are presented in this section. Figure A5 demonstrates the soil stabilized with WPFA and W1. The TGA analysis only showed a peak of calcite. Figure A6, Figure A7 and Figure A8 also demonstrated the same results for soil stabilized with WPFA or cement at different temperatures or sulfate concentrations.

### Appendix A.3. FTIR Results

The FTIR characterization of the soil stabilized with WPFA or cement is described in depth in this appendix. The full FTIR spectra of soil stabilized with WPFA and W1 is shown in Figure A9. The identified peaks are similar to those with W2, with difference of some lower intensities in some cases (normally the SO_4_ range is lower than those with W2).

The associated SO_4_ peaks for W3 (Figure A10) are similar to those with W2 and with same intensity except peak at 1430 cm^−1^. For the cement samples, these peaks are quite similar as identified for Figure A11 and Figure A12.
